# Inhibitory responses to retinohypothalamic tract stimulation in the circadian clock of the diurnal rodent *Rhabdomys pumilio*

**DOI:** 10.1096/fj.202200477R

**Published:** 2022-07-22

**Authors:** Robin A. Schoonderwoerd, Pablo de Torres Gutiérrez, Ruben Blommers, Anouk W. van Beurden, Tineke C. J. J. Coenen, Nathan J. Klett, Stephan H. Michel, Johanna H. Meijer

**Affiliations:** ^1^ Department of Cell and Chemical Biology Leiden University Medical Center Leiden The Netherlands; ^2^ Central Animal Facility Leiden University Medical Center Leiden The Netherlands

**Keywords:** calcium imaging, diurnality, GABA, glutamate, suprachiasmatic nucleus

## Abstract

In both diurnal and nocturnal mammals, the timing of activity is regulated by the central circadian clock of the suprachiasmatic nucleus (SCN). The SCN is synchronized to the external light cycle via the retinohypothalamic tract (RHT). To investigate potential differences in light processing between nocturnal mice and the diurnal rodent *Rhabdomys pumilio*, we mimicked retinal input by stimulation of the RHT ex vivo. Using Ca^2+^ imaging, we observed excitations as well as inhibitions of SCN neurons in response to electrical RHT stimulation. In mice, the vast majority of responses were excitatory (85%), whereas in *Rhabdomys*, the proportion of excitatory and inhibitory responses was similar (51% excitatory, 49% inhibitory). Glutamate blockers AP5 and CNQX blocked the excitatory responses to RHT stimulation but did not abolish the inhibitory responses in mice or *Rhabdomys*, indicating that the inhibitions were monosynaptically transmitted via the RHT. Simultaneous application of glutamate blockers with the GABA_A_ antagonist gabazine blocked all inhibitory responses in mice, but not in *Rhabdomys*. Collectively, our results indicate that in *Rhabdomys*, considerably more inhibitory responses to light are present and that these responses are driven directly by the RHT. We propose that this increased proportion of inhibitory input could reflect a difference in the entrainment mechanism employed by diurnal rodents.

**Abbreviations**
3V: third ventricle
ACSF: artificial cerebrospinal fluid
AOI: area of interest
AP5: amino‐5‐phosphonopentanoic acid
AUC: area under the curve
*bl*
: baseline values
CNQX: cyanquixaline
GABA: γ‐aminobutyric acid
GZ: gabazine
*K*_*d*_
: dissociation constant
OC: optic chiasm
PACAP: Pituitary adenylate cyclase‐activating polypeptide
RHT: retinohypothalamic tract
ROI: region of interest
SCN: suprachiasmatic nucleus
SD: Sprague–Dawley
*t*_*e*_
: transient end time
*t*_*m*_
: time to maximum/minimum
*t*_*s*_
: transient start time
ZT: Zeitgeber time

## 1 INTRODUCTION

The rotation of the Earth around its axis is driving 24‐h rhythms in the environment. To anticipate changes during the day and the night, nearly all species on the planet have developed an internal clock that allows them to time their activity relative to the light–dark cycle.^1^ Some species are predominantly active during the night (nocturnal), while others are predominantly active during the day (diurnal species). The mammalian central circadian clock is located in the suprachiasmatic nucleus (SCN) of the hypothalamus in both diurnal^2^ and nocturnal species.^3^ In all investigated species, the SCN is active during the day with respect to the electrical activity rhythm,^4^, ^5^ overall metabolism,^6^ and clock gene expression.^7^, ^8^, ^9^, ^10^ This has led to the assumption that diurnality and nocturnality are encoded by a sign reversal downstream of the SCN.^11^, ^12^ Despite the similarities among day‐active and night‐active species on the SCN tissue level, the neuronal wiring, including input and output organization, has been underinvestigated.

The SCN receives light input via the retinohypothalamic tract (RHT) for synchronization to the environmental cycle.^13^, ^14^, ^15^ While the pacemaker functions of the SCN are likely similar, there are indications that the light responsiveness of SCN neurons could differ between diurnal and nocturnal species. In nocturnal rats and hamsters, the vast majority of light‐responsive SCN neurons respond with excitations,^16^, ^17^ whereas neurons in the SCN of diurnal squirrels and degus show more inhibitions to light.^17^, ^18^ These findings challenge the view that the output from the central clock can fully explain differences in the temporal niche. So far, only a small number of diurnal species have been investigated, therefore, it remains unknown whether such differences reflect inter‐species variation, or whether they can be attributed more generally to diurnality and nocturnality.

For this purpose, we subjected diurnal *Rhabdomys pumilio* ^19^, ^20^ and nocturnal C3H mice (*Mus musculus*) to the same experimental protocol to compare the proportions of light‐responsive SCN cells. We electrically stimulated the RHT in hypothalamic slices containing the SCN to simulate light input,^21^ while we recorded the intracellular calcium concentrations under different pharmacological conditions. As a main result, we found that *Rhabdomys* have a significantly larger proportion of inhibitory light responses compared to mice and that these are driven monosynaptically from the RHT. This empowers the hypothesis that light inhibitions in the diurnal SCN are reflective of an entrainment mechanism involved in diurnal animals, and shows that an enhanced number of γ‐aminobutyric acid (GABA)ergic inputs are responsible for this.

## 2 MATERIALS AND METHODS

### 2.1 Animals

For this study, we used male C3H mice (Envigo, Horst, The Netherlands; 1–2 mo old; *n* = 11), obtained from Envigo labs, and male *Rhabdomys pumilio* (1–10 mo old; *n* = 6) from our breeding colony (breeding pairs were kindly provided to us by the lab of R.J. Lucas, Manchester University, UK). All animals were housed in controlled conditions, on a 12:12 h light–dark cycle with ad libitum access to food and water. The experiments were performed in accordance with the Dutch law on animal welfare and have been approved by the ethical committee of Leiden University Medical Center.

### 2.2 Slice preparation

**FIGURE 1:**
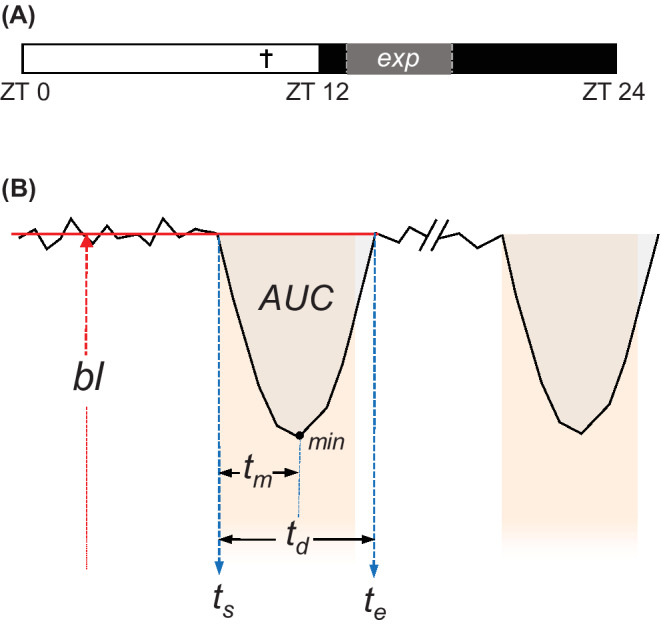
Experimental design and [Ca^2+^] transient analysis. (A) ZT0 to ZT12 constitutes the subjective day, ZT12‐ZT24 the subjective night. † = time of decapitation (ZT10). exp = time of recording and RHT stimulation. (B) Visualization of quantification measures: *bl*, baseline. *t*_*s*_, transient start time. *t*_*e*_, transient end time. *t*_*d*_, transient duration. *t*_*m*_, time to minimum. AUC, area under the curve. The colored background indicates the duration of electrical stimulation. For analysis, the parameters of both pulses are averaged for each cell and for each treatment.

At Zeitgeber Time (ZT) 10, in which ZT0 corresponds to the time of lights‐on and ZT12 to the time of lights‐off, the animals were anesthetized using 4% isoflurane gas (Isoflutek 1000) and decapitated (Figure 1A). The brain was extracted and stirred for a minute in ice‐cold modified artificial cerebrospinal fluid (ACSF) solution containing (in mM): NaCl 116.4, KCl 5.4, NaH_2_PO_4_ 1.0, MgSO_4_0.8, CaCl_2_1.0, MgCl_2_4.0, NaHCO_3_ 23.8, glucose 15.1 and 5 mg/L gentamicin (Gibco) saturated with 95% O_2_—5% CO_2_ (pH = 7.2–7.4, 290–310 mOsm). The brain was trimmed, mounted on a metal plate with super glue (Tube 3 G, Bison), and submerged in ice‐cold oxygenated modified ACSF. Three hundred microliters‐thick coronal slices were obtained using a vibrating‐blade microtome (VT 1000S, Leica Microsystems, Wetzlar, Germany). The slices were subsequently collected and maintained in an oxygenated regular ACSF solution containing (in mM): NaCl 116.4, KCl 5.4, NaH_2_PO_4_ 1.0, MgSO_4_0.8, CaCl_2_1.8, NaHCO_3_ 23.8, glucose 15.1, and 5 mg/L gentamicin (Gibco) saturated with 95% O_2_—5% CO_2_ (pH = 7.2–7.4, 290–310 mOsm). The slices were incubated for 20 min at 37°C, followed by a 1‐h incubation at room temperature. Oxygen and CO_2_ were supplied into the solution at all times.

### 2.3 Ca^2+^ imaging

Hypothalamic slices (*n* = 16 for C3H mice; *n* = 10 for *Rhabdomys*) containing the SCN were loaded with the Ca^2+^ indicator dye Fura‐2‐acetoxymethyl ester (Fura‐2 AM; AAT Bioquest, Sunnyville, CA, USA) as based on previously described methods.^22^ The Fura‐2AM fluorescence excitation spectrum is sensitive to changes in Ca^2+^ concentration. Slices were incubated at room temperature for 1 h in Fura‐2AM (7.92 μM) diluted in oxygenated ACSF. Subsequently, the slices were washed four times with oxygenated ACSF. All steps involving Fura‐2AM were performed under dim light to prevent photobleaching.

**FIGURE 2:**
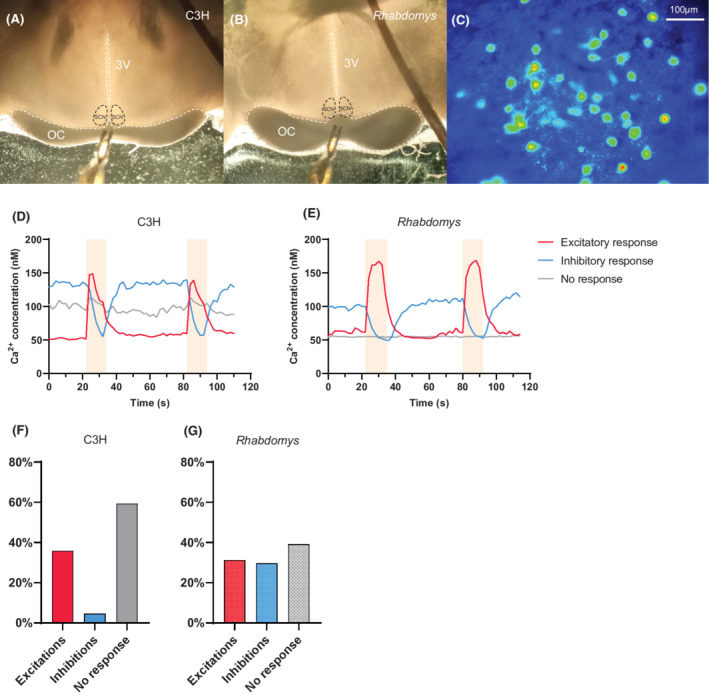
Electrical stimulation of the RHT causes excitations and inhibitions in SCN neurons. (A) Localization of the electrode in the optic chiasm in C3H mice. (B) *Rhabdomys*. OC, optic chiasm; 3 V, third ventricle. (C) Example image of Fura‐2 AM fluorescence at the border of the optic chiasm and SCN. (D) Representative raw traces from individual cells showing excitations, inhibitions, and no response to RHT stimulation in C3H mice. (E) *Rhabdomys*. The colored background indicates the duration of stimulation. (F) Proportions of each response type for C3H mice and (G) *Rhabdomys*.

SCN brain slices were positioned in a recording chamber that was mounted on the fixed‐stage of an upright fluorescence microscope (Axioskop 2‐FS plus, Carl Zeiss Microimaging, Oberkochen, Germany). A constant flow of oxygenated ACSF (1.5–2 ml/min) was delivered to the chamber using a valve‐controlled gravity perfusion system (ALA Scientific Instruments BPS‐4) and suction was generated by a vacuum pump. The Ca^2+^ indicator dye was excited alternatively at wavelengths of 340 and 380 nm with a monochromator (Polychrome V, TILL Photonics, Germany), and the emission light (505 nm) was captured by a camera mounted on the microscope (PCO Imaging Sensicam, TILL Photonics; 50 ms exposure time, 300 ms live cycle time, field of view: 688 × 520 μm). Images were acquired every 2 s and recorded by TILLVision software (TILL Photonics). Electrical stimulation of the RHT was achieved by placing a custom‐built bipolar electrode (Pt/Ir, Teflon coated, Ø50 μm) in the center of the optic chiasm (Figures 2A,B). The tips of the electrode were placed in the optic chiasm, perpendicular to the third ventricle. A stimulator (Grass Instruments S88 Dual Output Square Pulse Stimulator, Grass SIU5 Stimulus Isolation Unit; or Neuro Data PG4000 Digital Stimulator) was used to generate pulses (3–4 V, 20 Hz, 4 ms, 10s pulse duration). The optimal voltage was determined empirically before the start of experimentation. Each Ca^2+^ recording lasted about 120 s and involved the delivery of two electrical pulses 60s apart from each other. The responses to the two pulses were averaged in subsequent analyses. The experiments entailed five sequential measurements that differed in the composition of the medium being perfused into the recording chamber (all the drugs were water‐soluble): (1) ACSF; (2) D‐amino‐5‐phosphonopentanoic acid (AP5, 50 μM; Tocris Bioscience) and cyanquixaline (CNQX, 20 μM; Tocris Bioscience) in ACSF; (3) d‐AP5 (50 μM,) CNQX (20 μM), and gabazine (GZ, 10 μM; Tocris Bioscience) in ACSF; (4) ACSF as drug washout; (5) Negative control with modified ACSF (high Mg^2+^). The solutions were actively bubbled with oxygen and perfused in the recording chamber for 10 min prior to the start of the imaging sessions. All recordings were performed between ZT13 and ZT17 (Figure 1A).

### 2.4 Data analysis

Ca^2+^ imaging data were analyzed with the software TILLVision and Python version 3.0.9. For every recording, cells were encircled to select regions of interest (ROIs), and an area of interest (AOI) devoid of cells was chosen as a background. The averaged fluorescence intensity within each ROI and AOI for either the 340 or 380 nm channel for a specific time point was computed as a numerical value. The mean ROI 340 and 380 nm fluorescence values were corrected by subtracting the corresponding background fluorescence and used to calculate Fura‐2AM excitation ratios for every time point following the formula: $\;r=\frac{F_{340}}{F_{380}}\;$.

Approximate Ca^2+^ concentrations for every ratio value were then calculated according to the formula: $\left[{\mathrm{Ca}}^{2+}\right]=\beta \cdot K_{d}\cdot \frac{r-r_{\min}}{r_{m\mathrm{ax}}-r}$, with a *β* of 7.48, and a dissociation constant (*K*_*d*_) of 230 nM. r_min_ and r_max_ corresponded to the minimum and maximum ratio values observed, respectively. The constants were experimentally determined by calibration prior to the start of the experiments (*r*_min_ = 149 and *r*_max_ = 5262). Cells with extreme basal [Ca^2+^] values (above 300 nM) were excluded from the analysis. A graphical representation of the 340 and 380 values was obtained, as well as Ca^2+^ concentration values across the experiment. Additionally, we devised an algorithm to analyze the resulting [Ca^2+^] transients. Baseline values (*bl*) were calculated by averaging [Ca^2+^] values 20s prior to stimulation pulses. Next, the transient start (*t*_*s*_) and end times (*t*_*e*_) were assessed to obtain transient duration *(t*_*d*_; *t*_*d*_ = *t*_*e*_ − *t*_*s*_). *t*_*s*_ was set as the start of the stimulation pulses, whereas *t*_*e*_ was set as the timepoint in which [Ca^2+^] values returned to its corresponding baseline ±10% (Figure 1B). In two instances where [Ca^2+^] did not return to baseline levels, the stimulation was excluded from the analysis. The time in between *t*_*s*_ and the highest or lowest point of the transient was denoted as *t*_*m*_. The area under the curve (AUC) was computed after using the trapezoidal rule and correcting for the baseline (*t*_*d*_ · *bl*), resulting in positive values for upwards [Ca^2+^] transients and negative values for downwards [Ca^2+^] transients (Figure 1B). In addition to these quantitative outcomes, a qualitative analysis of the [Ca^2+^] transients was performed to categorize each [Ca^2+^] transient as excitatory, inhibitory, or no response. For the qualitative analysis, AUC measurements were standardized for their baseline values and the duration of the transients according to the formula: $\text{Normalized area}=\frac{\mathrm{AUC}}{\mathrm{bl}∙t_{d}}$. Threshold values of −0.1 and 0.1 were selected to qualitatively distinguish between inhibitory, excitatory, and no responses, corresponding to a 10% decrease or a 10% increase in [Ca^2+^], respectively. For AUC representation in violin plots, cells were grouped based on the response type in the control situation. Outliers identified using the ROUT outlier removal method in PRISM Graphpad (with a maximum false discovery rate of 1%) were excluded from visualization.

Responses were only included if the following criteria were met: (1) [Ca^2+^] transients had the same response type for both stimulations; (2) The response was attenuated after incubating with a high [Mg^2+^] solution. A response was considered biphasic if, during the duration of *t*_*d*_, values both above and below ±10% of the baseline were detected.

### 2.5 Statistical analysis

Data were analyzed using SPSS 25 software (IBM. Armonk, NY, USA). The number of excitatory, inhibitory, and no responses across C3H and *Rhabdomys* was compared with a Chi‐squared test. A linear mixed model was used to determine the effect of the drugs where the AUC was the dependent variable. The condition (ACSF control, AP5 + CNQX, AP5 + CNQX+GZ), type of response during the control (excitatory, inhibitory, no response), and their interaction were included as fixed factors of the analysis. The covariance matrix was assumed to be diagonal. A random intercept was included, and the subject groupings considered were the animals and slices to the cells belonged. A post hoc analysis was carried out with pairwise comparisons with Bonferroni adjustment to correct for multiple comparisons. The significance level was set at *α* = 0.05.

## 3 RESULTS

### 3.1 RHT stimulation elicited both excitatory and inhibitory responses in SCN neurons

Hypothalamic slices (300 μm thick) containing the SCN were stained with a calcium dye (Fura‐2AM; Figure 2C). To simulate light input to the SCN, the RHT was electrically stimulated with a bipolar electrode (Figure 2A,B). The resulting calcium transients were measured in nocturnal C3H mice (*n* = 360 cells) and the diurnal rodent *Rhabdomys pumilio* (*n* = 362 cells).

Upon electrical stimulation, three different outcomes could be distinguished: excitatory responses, inhibitory responses, and non‐responders. Examples are shown for C3H (Figure 2D) and *Rhabdomys* (Figure 2E). Baseline Ca^2+^ values preceding RHT stimulation were compared between all groups using the Kruskal‐Wallis test. Post‐hoc Dunn's test revealed only a significant difference between the baselines of non‐responders and cells showing inhibitory responses in *Rhabdomys* (*p* = .008; Supporting Information Figure S1). Proportions of the different response types differed between species. Of the responsive cells in C3H mice, 129 out of 146 (88.4%) cells demonstrated an increase in intracellular calcium, while 17 out of 146 (11.6%) demonstrated a decrease in intracellular calcium (Figure 2F). In *Rhabdomys* responsive SCN cells, 113 out of 220 (51.4%) responses were excitatory while 107 out of 220 (48.6%) responses were inhibitory (Figure 2G). The proportions of response types are significantly different between C3H mice and *Rhabdomys* (*χ* ^2^ = 53.61; *p* < .001; Chi‐squared test). The proportions of response types are shown for different SCN regions separately in Supporting Information Figure S2.

### 3.2 Responses were modulated by glutamate and GABA blockers

To determine whether the cellular responses were mediated by glutamate, the RHT stimulation was repeated while glutamatergic neurotransmission was blocked using AP5 and CNQX. Among the responses resistant to glutamate receptor blockers, the GABA_A_‐mediated responses were identified by adding the GABA_A_ receptor blocker gabazine (GZ) to the AP5 + CNQX mix. GABA_B_ receptor antagonists were not used, as these affect glutamate release from the RHT.^23^ The effect of drug administration was quantified for 360 cells from C3H mice and 260 cells from *Rhabdomys* (from the same sample as in Figure 2) by calculating the area under the curve (AUC) in the [Ca^2+^] traces. AUC values for the three different response types (excitatory, inhibitory, or non‐responder, as defined in the control condition) were analyzed for each pharmacological condition. For excitatory responses, blocking glutamate transmission would be expected to diminish the responses. Indeed, AP5 + CNQX resulted in a significant decrease in response size, reflecting the transition from responding with excitations to non‐responsiveness.

**FIGURE 3:**
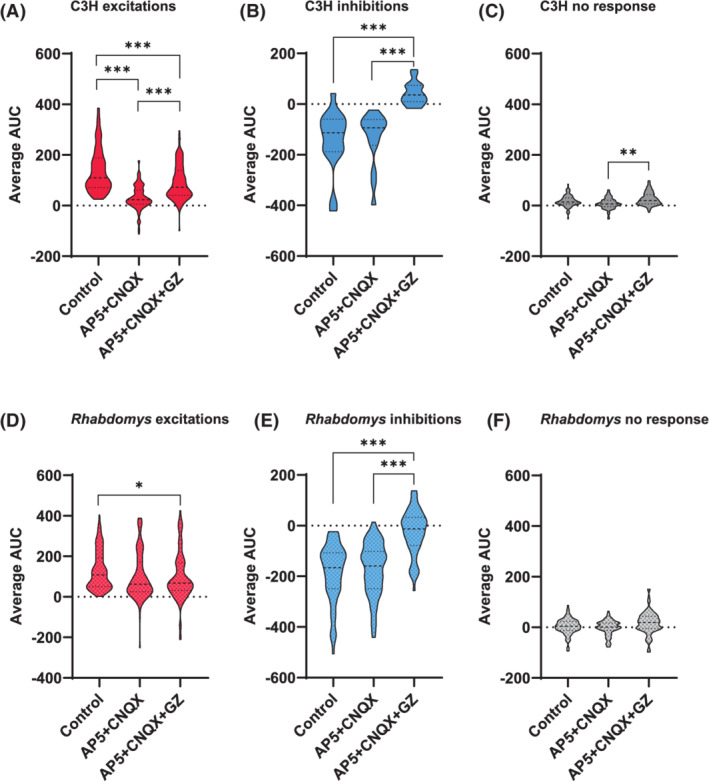
Quantitative responses to pharmacological blockade of glutamatergic and GABAergic signaling. Violin plots showing the quantified response to RHT stimulation. (A) C3H neuron responses separated by response type in the control situation: (A) excitations, (B) inhibitions, (C) non‐responders, (D) same for *Rhabdomys* excitations, (E) inhibitions, and (F) non‐responders. *p < .05, **p < .01, ***p < .001.

In C3H mice, excitatory Ca^2+^ transients were significantly different in all three drug conditions (*p* < .001; linear mixed model analysis; Figure 3A). For inhibitory responses, blocking glutamate transmission might be expected to have no effect on AUC size, in contrast to blocking GABA. Indeed, the inhibitory responses in AP5 + CNQX+GZ condition were significantly smaller compared to the control and AP5 + CNQX conditions (both *p* < .001; Figure 3B). Surprisingly, after applying GZ, the non‐responder neurons demonstrated an increase in AUC size in the presence of gabazine (AP5 + CNQX+GZ) compared to the AP5 + CNQX condition (*p* = .002; Figure 3C).

In *Rhabdomys*, glutamate blockade (AP5 + CNQX) did not significantly change the average size of excitatory responses, but when gabazine was applied in conjunction with glutamatergic blockade (AP5 + CNQX+GZ), the average response size was reduced compared to control (*p* = .017; Figure 3D), suggesting an involvement of GABA‐mediated excitations. The pattern of drug effect for inhibitory responses in *Rhabdomys* was similar to that observed in C3H mice, with the AP5 + CNQX+GZ condition yielding smaller responses compared to the control or glutamate‐blockade condition (both *p* < .001; Figure 3E). Non‐responders showed no difference between conditions (Figure 3F).

### 3.3 Heterogeneity in response patterns after neurotransmitter blocker application

**FIGURE 4:**
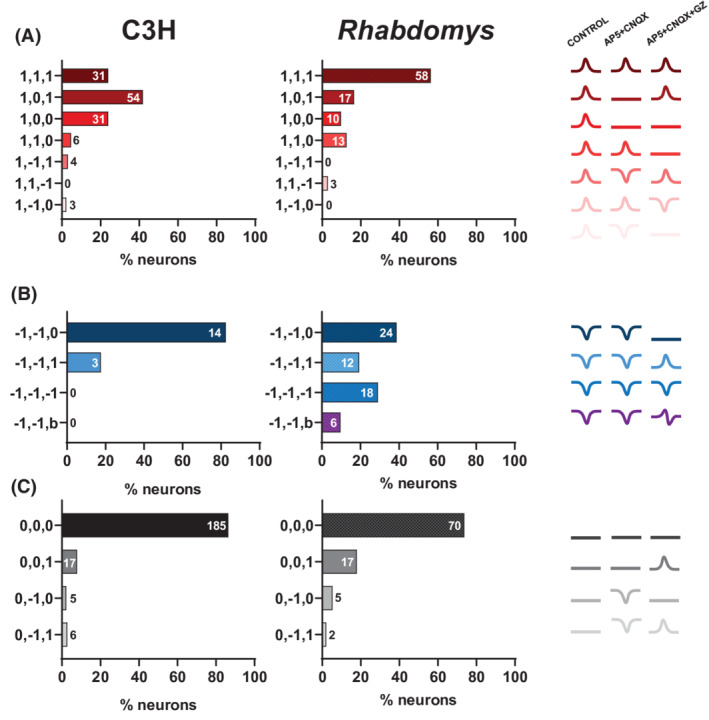
Qualitative response dynamics categorization. (A) Excitations, (B) inhibitions, and (C) non‐responders classified by their response over the three conditions (control, AP5 + CNQX and AP5 + CNQX+GZ). Only response categorizations with more than one cell following those dynamics were included in the graphical representation. The numbers at the y axis represent the responses in the three conditions (1 = excitation, −1 = inhibition, 0 = no response, b = biphasic). Results are sorted by the most prevalent response pattern. The numbers inside the bars represent the number of cells displaying this response pattern.

Response dynamics for all cells were categorized based on their response in all three conditions. In C3H mice, 85 out of 129 (65.9%) excited cells lost the response upon AP5 + CNQX treatment. This occurred in 27 out of 103 (26.2%) light‐excited *Rhabdomys* cells. In 6 out of 129 (4.7%) light‐excited cells in C3H mice and 13 out of 103 (12.6%) excited *Rhabdomys* cells, the excitations were abolished by AP5 + CNQX+GZ treatment, hinting at excitatory responses to GABA. However, in C3H mice and *Rhabdomys*, 31 out of 129 (24.0%) and 58 out of 103 (56.3%) excitatory responses, respectively, remained excitatory in the different pharmacological conditions, suggesting that the glutamate blockers did not block all excitations (Figure 4A). In C3H mice, all inhibitory responses were abolished or inverted after treatment with AP5 + CNQX+GZ. For *Rhabdomys*, however, two additional inhibitory response patterns were observed. Upon AP5 + CNQX+GZ treatment, 29.0% of *Rhabdomys* cells remained inhibitory, and 9.7% of responses turned biphasic (Figure 4B). This suggests that apart from GABA, another inhibitory mechanism is involved. Of the non‐responding cells, the vast majority (86.4% for C3H mice and 73.7% for *Rhabdomys*) remained non‐responders (Figure 4C). Representative traces of cells demonstrating different response patterns to receptor blockade are provided in Supporting Information Figures S3 and S4 for C3H mice and *Rhabdomys*, respectively.

### 3.4 Prolonged GZ‐insensitive inhibitions in the *Rhabdomys* SCN

**FIGURE 5:**
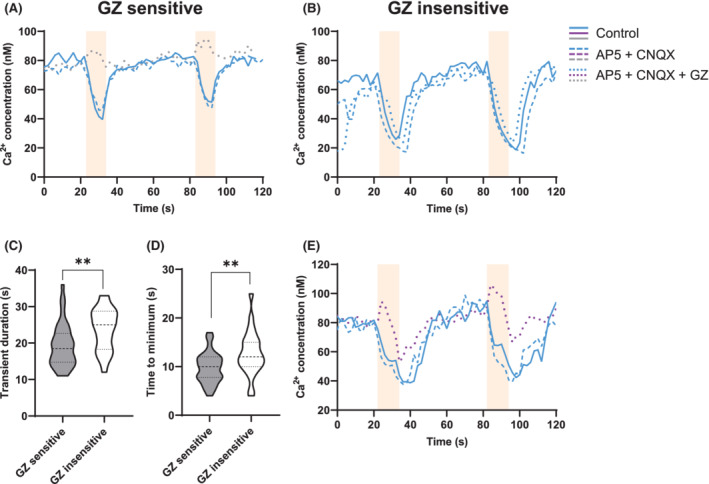
Prolonged non‐GABAergic inhibitions in *Rhabdomys*. (A) Example traces of a gabazine‐sensitive neuron. (B) A gabazine‐insensitive neuron. (C) Violin plots of the average timing of the minimum [Ca^2+^]. (D) transient duration for the gabazine‐sensitive and insensitive neurons. (E) Example of a two‐component cell, showing a partly gabazine‐sensitive and partly gabazine‐insensitive response.

In contrast to inhibitory responses in C3H mice, which were all blocked by gabazine (Figures 3B and 4B), only some of the inhibitory responses in *Rhabdomys* were blocked. As compared to the inhibitory responses that were blocked by gabazine (*n* = 38; Figure 5A), the cells that were not blocked by gabazine (*n* = 24, Figure 5B) showed longer [Ca^2+^] transient duration (*t*_*d*_; *p* = .005; Student's *t*‐test), reaching their minimum (*t*_*m*_) on average after the 10‐s stimulation window (12.8 s after stimulation onset; Figure 5C), and slower recovery to baseline values (21.2 s; *p* = .012; Student's *t*‐test; Figure 5D). This suggests that the—yet undefined—inhibitory mechanism has slower response dynamics. Some cells were observed in which two different components of inhibition were observed. An initial GABAergic component was either blocked or reversed by gabazine, and a second component that was unaffected by gabazine retained its prolonged inhibitory dynamic resulting in a biphasic response (Figure 5E).

## 4 DISCUSSION

Light is the most important factor to entrain the circadian clock and understanding how light is processed in the diurnal SCN is critical to translate circadian clock research from nocturnal rodents to humans. Previous studies found an increased proportion of inhibitory light responses in SCN neurons of two diurnal rodent species.^17^, ^18^ We extend these findings by characterizing the responses in yet another diurnal rodent: *Rhabdomys pumilio*.

**FIGURE 6:**
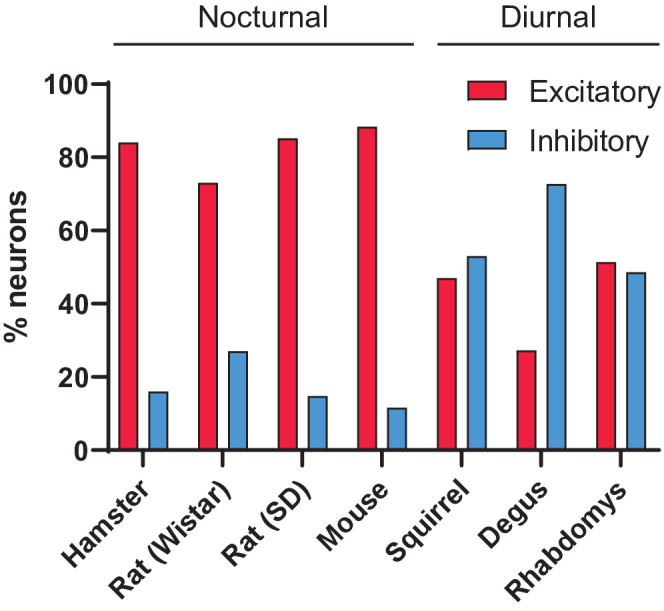
Proportion of light‐excited and light‐inhibited cells in the SCN of nocturnal and diurnal species. Hamster and rat (Wistar) are from Meijer et al.,^16^ rat (Sprague–Dawley) and degus data are from Jiao et al.,^17^ squirrel data are from Meijer et al.,^18^ and mouse and *Rhabdomys* data are from this study. Hamsters, rats, and mice are nocturnal species. Squirrels, degus, and *Rhabdomys* are diurnal species.

For this purpose, we performed calcium imaging experiments in SCN slices of the C3H strain of nocturnal mice and the diurnal rodent *Rhabdomys*. RHT stimulation resulted in excitations, inhibitions, and cells that showed no responses, in line with previous studies using this method.^24^ We found that the proportion of inhibitions in *Rhabdomys* was larger than that observed in C3H mice (49% and 12% of light‐responsive neurons, respectively). This result is in accordance with previous studies in diurnal squirrels^18^ and degus^17^; assayed with in vivo extracellular recording, these diurnal rodents showed inhibition following light exposure in 53% and 73% of light‐responsive cells, respectively. These findings are in stark contrast to those observed in nocturnal hamsters, Wistar rats^16^ and Sprague–Dawley rats,^17^ that exhibit 16%, 27%, and 15% of light‐inhibited cells, comparable to the 12% of inhibited cells observed in C3H mice in this study (Figure 6; *χ* ^2^ = 125.61; *p* < .001; Chi‐squared test). Additional evidence for light‐inhibition in the diurnal SCN can be found in humans using functional magnetic resonance imaging (fMRI).^25^ Although it is not possible to measure responses from individual neurons with fMRI, the tissue‐level blood‐oxygen‐level‐dependent (BOLD) signal showed reduced BOLD signal in the SCN following light exposure.

Up until recently, models posited that the RHT only contains excitatory projections to the SCN.^26^ In this case, all SCN responses to RHT stimulation would be predicted to have been abolished following AP5 + CNQX incubation. However, in both C3H mice and *Rhabdomys*, we continued to observe inhibitions in the presence of AP5 and CNQX. This is in line with previous studies in mice, rats, and degus,^27^, ^28^ and suggests that the RHT contains inhibitory GABAergic fibers that project to the SCN.

The functional role of RHT inhibitory input is not known, and therefore, it is unclear how inhibition is involved in entrainment or related to diurnality. A recent study performed in nocturnal mice suggested that inhibition via retinal GABAergic input dampens the light sensitivity of the circadian clock, thereby shifting the dynamic range of photoentrainment to higher light intensities.^28^ An increased proportion of inhibition in the diurnal SCN could reflect the increased need to dampen light sensitivity, as diurnal species are generally exposed to higher light intensities than nocturnal species.

Alternatively, the light‐inhibited subpopulation of SCN neurons could function as a gate for the acute effects of light in the non‐image‐forming visual system. Indeed, several lesion studies indicate that the SCN is essential for conveying the light signal that mediates melatonin suppression,^29^ glucocorticoid release,^30^ and behavioral masking.^29^, ^31^ Importantly, for glucocorticoid release^32^, ^33^, ^34^, ^35^ and behavioral masking, the response to light in diurnal species is opposite to that in nocturnal animals.^36^ Therefore, it is possible that these important differences are already signaled by the RHT. If so, the same neurons that act as pacemaker neurons could have a secondary function in mediating the effects of light in non‐visual processes.

Interestingly, we found evidence that, besides GABA_A_ signaling, another form of inhibition is encoded in the SCN of *Rhabdomys*. Whereas 100% of inhibitions observed in C3H mice were blocked with GZ, only 36 out of 62 (58.1%) inhibitions were blocked in *Rhabdomys*. The time course of GABAergic responses in *Rhabdomys* was similar to those recorded in C3H mice. However, the inhibitory responses that were not blocked by GZ (which were only observed in *Rhabdomys*), were longer in duration. GZ‐sensitive and—insensitive cells coexisted in the same slice recordings, confirming that the GZ was effective in these experiments. Additionally, 6 out of 24 (25.0%) persistent inhibitions appeared to consist of a GABAergic and a non‐GABAergic component and showed biphasic responses in the AP5 + CNQX+GZ condition. Recently, a patch‐clamp study in *Rhabdomys* SCN slices reported a delayed recovery of firing after a hyperpolarizing current injection.^37^ This delay has not been previously identified in nocturnal species and therefore may reflect an intrinsic characteristic of the diurnal SCN. The prolonged inhibitory responses observed in our study might be related to this delayed recovery. As GZ blocks only GABA_A_ receptors, it is possible that GABA_B_ receptors mediate the GZ‐insensitive inhibitions. Alternatively, a different inhibitory neurotransmitter or neuromodulator could be present in the diurnal SCN. Slow responses are commonly observed following G‐protein‐coupled receptor activation^38^ by neuropeptides such as pituitary adenylate cyclase‐activating polypeptide (PACAP).^39^, ^40^ Thus, the kinetics of the GZ‐insensitive responses observed suggest an involvement of a neuropeptide.

In 34 out of 307 (11.1%) non‐responsive cells, excitatory responses were revealed after the application of AP5 + CNQX+GZ. This can be explained by neurons receiving both inhibitory and excitatory input. With the loss of inhibitory input through the action of GZ, [Ca^2+^] response elevations would then be unmasked.^24^ In both C3H mice and *Rhabdomys*, a subset of excitatory responses was not blocked with the combination of AP5 and CNQX. This is rather unexpected, as most excitations are presumed to be caused by glutamate release from the RHT. A proportion of the excitations in the AP5 + CNQX condition can be explained by the excitatory actions of GABA. Indeed, GABAergic excitation has been reported to occur in retinorecipient cells of the SCN.^41^ However, it seems that the cells with persistent excitation in the presence of glutamatergic and GABAergic antagonists might be caused by alternative excitatory mechanisms, such as PACAP or metabotropic glutamate receptors. The non‐glutamatergic excitatory inputs and non‐GABAergic inhibitory inputs found in our data are suggestive of heterogeneity within SCN neurons.

In summary, we found a larger proportion of light‐inhibited cells in the *Rhabdomys* SCN compared to the mouse SCN. We showed that these inhibitions were elicited directly by RHT fibers. Furthermore, we found indications for a previously unidentified inhibitory neurotransmitter or neuropeptide that is present in the *Rhabdomys* RHT, but not in the murine RHT. These inhibitory mechanisms add to evidence that the diurnal SCN has different light‐response properties compared to the SCN of nocturnal species. Understanding the role of light‐inhibited SCN neurons in diurnal species will not only contribute to a better understanding of photic entrainment in diurnal animals but will also allow for more accurate translations of circadian research to humans.

## AUTHOR CONTRIBUTIONS

Robin A. Schoonderwoerd, Ruben Blommers, Stephan H. Michel and Johanna H. Meijer conceived the project and designed the experiments. Tineke C.J.J. Coenen was responsible for breeding the *Rhabdomys*. Robin A. Schoonderwoerd, Pablo de Torres Gutiérrez, Ruben Blommers, Nathan J. Klett and Stephan H. Michel conducted the experiments. Robin A. Schoonderwoerd, Pablo de Torres Gutiérrez, Ruben Blommers, Stephan H. Michel and Johanna H. Meijer analyzed the data. Robin A. Schoonderwoerd, Pablo de Torres Gutiérrez, Anouk W. van Beurden and Johanna H. Meijer prepared the figures and wrote the manuscript.

## FUNDING INFORMATION

ERC Advanced, Grant/Award number 834513; Velux Stiftung foundation, Grant/Award number 1131.

## DISCLOSURES

The authors declare no competing interests.

## Supporting information


Figure S1

Table S2

Data S3

## Data Availability

All datasets are included in the Supporting Information of this article.
