# Supplementary material for: Inhibitory responses to retinohypothalamic tract stimulation in the circadian clock of the diurnal rodent Rhabdomys pumilio
Source: FASEB J. 2022 Jul 22;36(8):e22415. doi: 10.1096/fj.202200477R (PMC9544711; doi:10.1096/fj.202200477R)
Supplement: Supplementary file 1 — Figure S1 [file FSB2-36-e22415-s002.docx]

## Supporting information


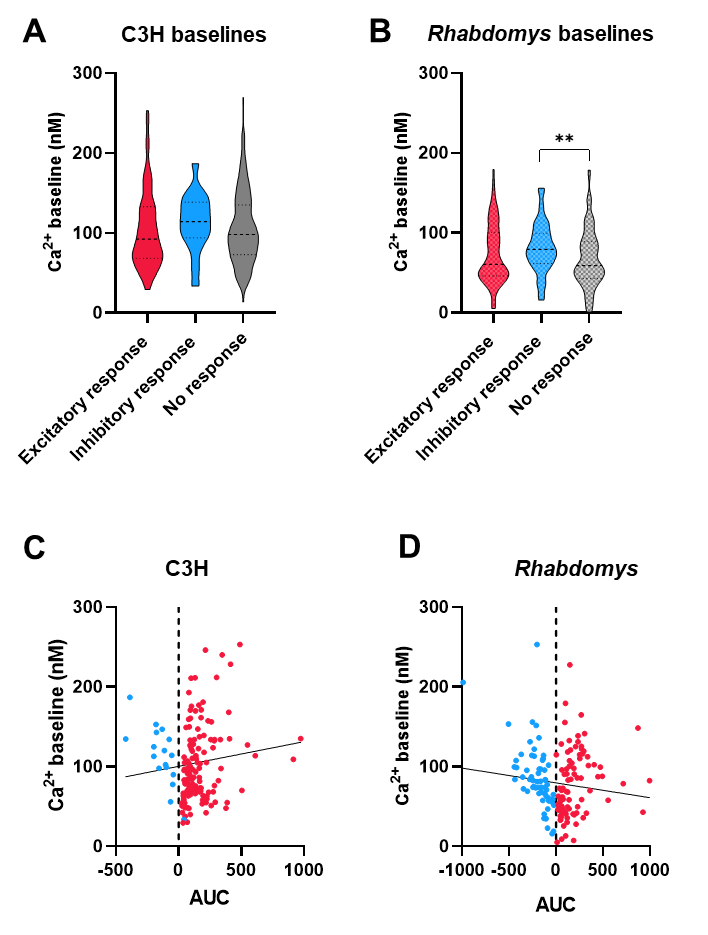


**Supplemental Figure S1: Violin plots of Ca^2+^ baseline values.** (A) For C3H mice and (B) *Rhabdomys* SCN cells, the Ca^2+^ baseline values preceding the RHT stimulation were calculated. (C) The Ca2+ baseline plotted verses the magnitude of the response for excitations (red) and inhibitions (blue) in C3H (slope *p* = 0.158) and (D) *Rhabdomys* (slope *p* = 0.144). ***p*<0.01


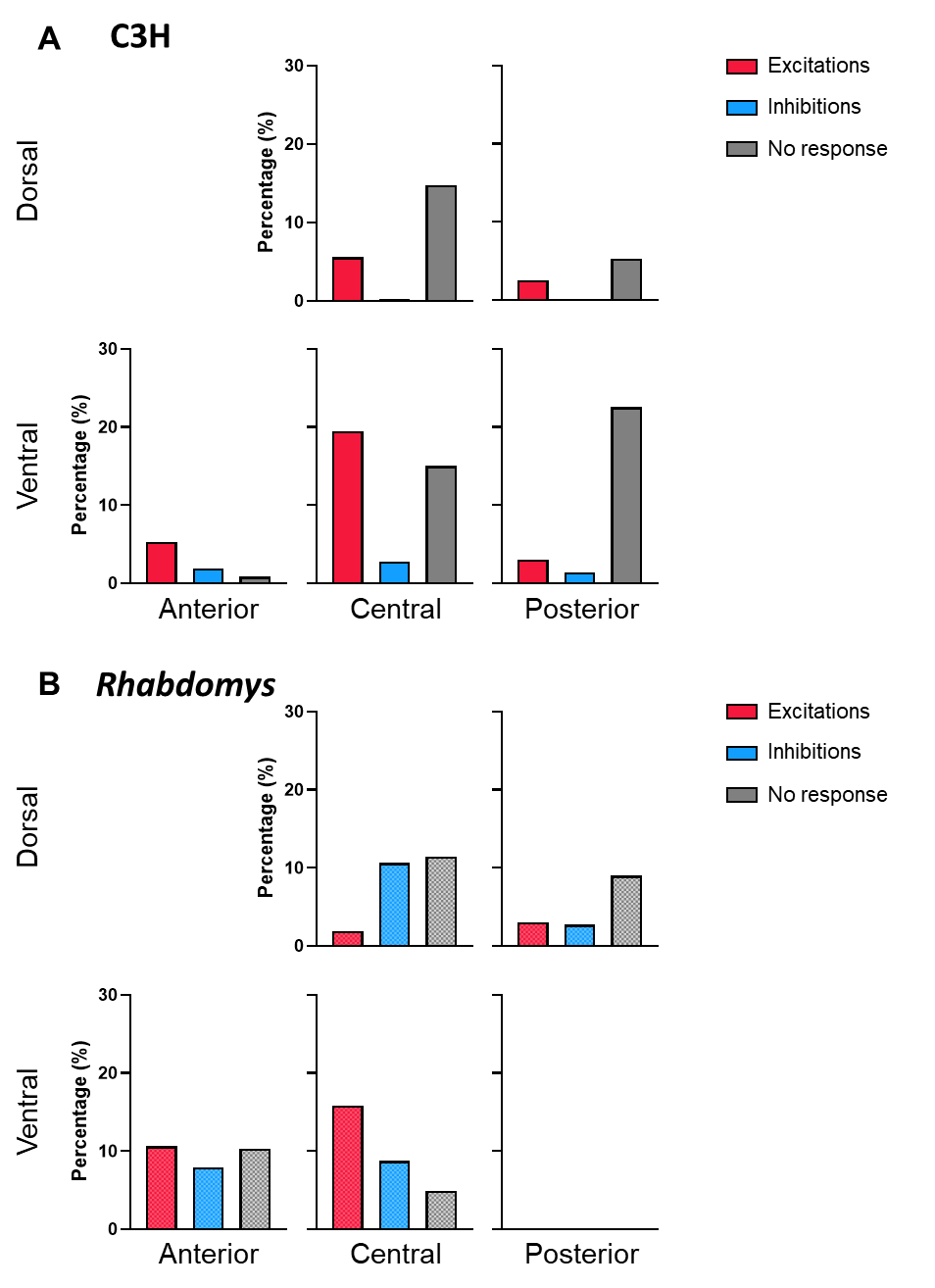


**Supplemental Figure S2: Proportion of response types for SCN regions separately.** (A) For both C3H mice and (B) *Rhabdomys,* the proportions of response types are separated by the location on the ventral-dorsal axis and on the anterior-posterior axis.


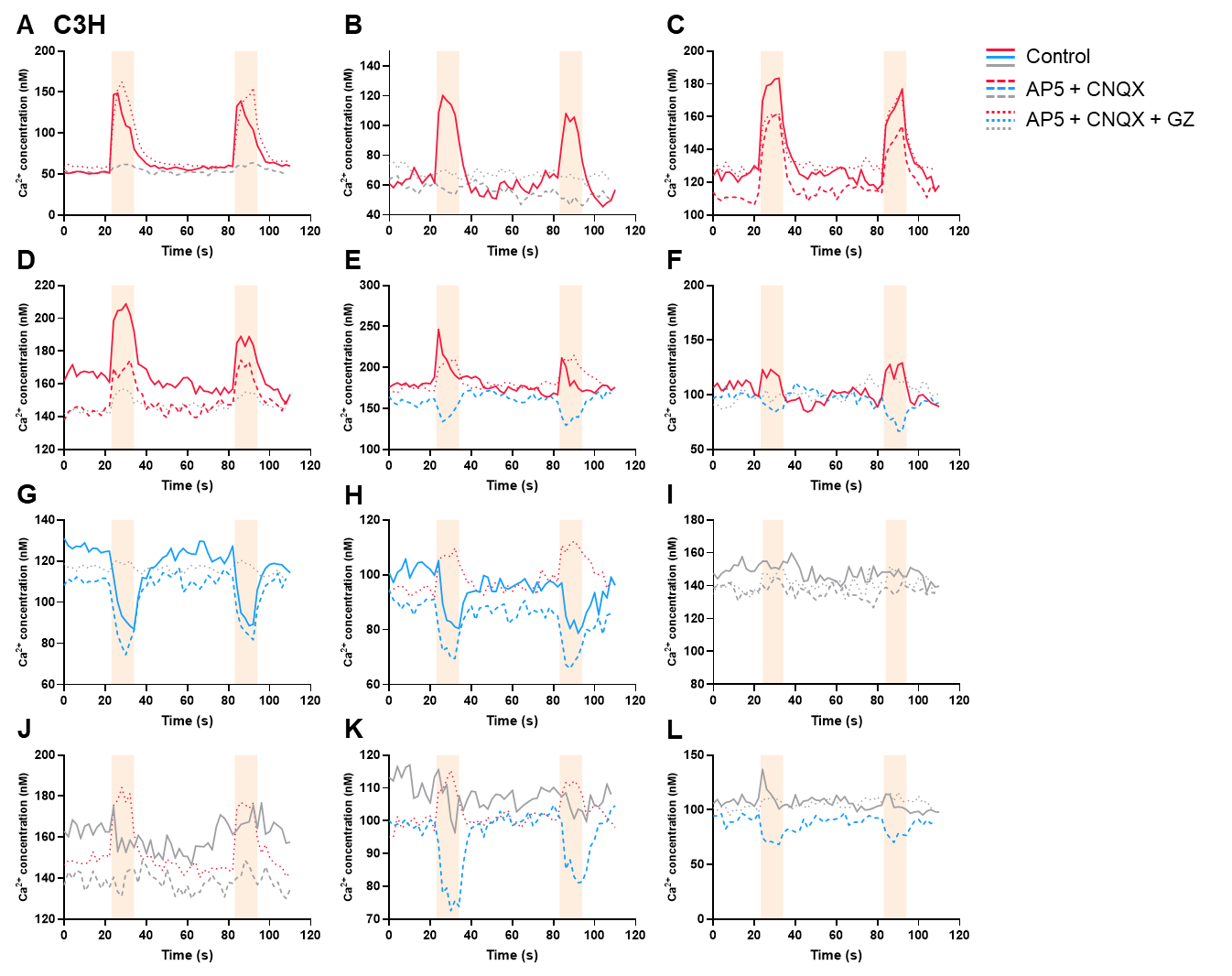


**Supplemental Figure S3: Example [Ca^2+^] traces of C3H mice cells*.*** Each graph corresponds to the responses of a single cell in the different pharmacological conditions. The solid line represents the control condition with only ACSF, a dashed line represents the AP5+CNQX condition and the dotted line represents the AP5+CNQX+GZ condition. Blue lines mark excitations, red lines inhibitions and grey lines non-responders. Examples are shown for all observed response patterns.


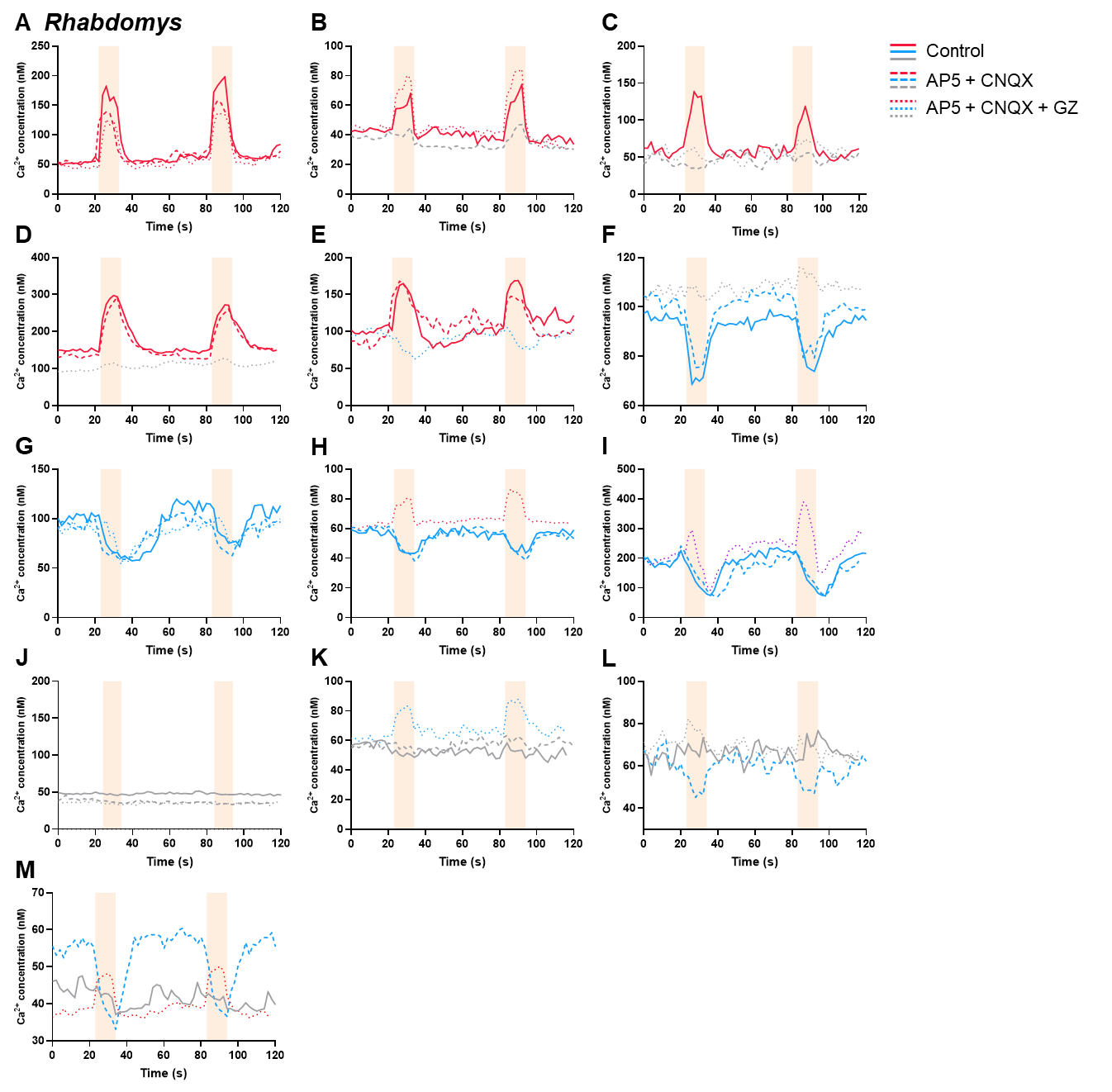


**Supplemental Figure S4: Example [Ca^2+^] traces of *Rhabdomys* cells*.*** Each graph corresponds to the responses of a single cell in the different pharmacological conditions. The solid line represents the control condition with only ACSF, a dashed line for the AP5+CNQX condition and the dotted line represents the AP5+CNQX+GZ condition. Blue lines mark excitations, red lines mark inhibitions and grey lines mark non-responders. Examples are shown for all observed response patterns.
